# Diagnostic accuracy of qPCR and microscopy for cutaneous leishmaniasis in rural Ecuador: A Bayesian latent class analysis

**DOI:** 10.1371/journal.pntd.0011745

**Published:** 2023-11-29

**Authors:** Jacob M. Bezemer, Joanna Merckx, Byron P. Freire Paspuel, Manuel Calvopiña, Henry J. C. de Vries, Henk D. F. H. Schallig, Mariska M. G. Leeflang, Nandini Dendukuri

**Affiliations:** 1 Hospital Shell, Fundación Misión Cristiana de Salud, Shell, Pastaza, Ecuador; 2 Department of Medical Microbiology and Infection Prevention, Laboratory for Experimental Parasitology, Amsterdam University Medical Centers location Academic Medical Center at the University of Amsterdam, Amsterdam, the Netherlands; 3 Amsterdam Institute for infection and Immunity, Infectious Diseases Program, Amsterdam, the Netherlands; 4 Department of Epidemiology, Biostatistics, and Occupational Health, McGill University, Montreal, Canada; 5 Department of Epidemiology and Data Science, Amsterdam UMC location University of Amsterdam, Amsterdam, the Netherlands; 6 Laboratorios de Investigación, Universidad de las Américas, Quito, Ecuador; 7 Vall d’Hebron Research Institute, Hospital Universitari Vall d’Hebron, Barcelona, Spain; 8 OneHealth Research Group, Facultad de Medicina, Universidad de las Américas, Quito, Ecuador; 9 Department of Infectious Diseases, Center for Sexual Health, Public Health Service, Amsterdam, the Netherlands; 10 Research Institute of the McGill University Health Centre, Montreal, Quebec, Canada; INGEBI, ARGENTINA

## Abstract

**Background:**

Clinical and laboratory diagnosis of cutaneous leishmaniasis (CL) is hampered by under-ascertainment of direct microscopy.

**Methods:**

This study compared the diagnostic accuracy of qPCR on DNA extracted from filter paper to the accuracy of direct smear slide microscopy in participants presenting with a cutaneous lesion suspected of leishmaniasis to 16 rural healthcare centers in the Ecuadorian Amazon and Pacific regions, from January 2019 to June 2021. We used Bayesian latent class analysis to estimate test sensitivity, specificity, likelihood ratios (LR), and predictive values (PV) with their 95% credible intervals (95%CrI). The impact of sociodemographic and clinical characteristics on predictive values was assessed as a secondary objective.

**Results:**

Of 320 initially included participants, paired valid test results were available and included in the diagnostic accuracy analysis for 129 from the Amazon and 185 from the Pacific region. We estimated sensitivity of 68% (95%CrI 49% to 82%) and 73% (95%CrI 73% to 83%) for qPCR, and 51% (95%CrI 36% to 66%) and 76% (95%CrI 65% to 86%) for microscopy in the Amazon and Pacific region, respectively. In the Amazon, with an estimated disease prevalence among participants of 73%, negative PV for qPCR was 54% (95%CrI 5% to 77%) and 44% (95%CrI 4% to 65%) for microscopy. In the Pacific, (prevalence 88%) the negative PV was 34% (95%CrI 3% to 58%) and 37% (95%CrI 3% to 63%). The addition of qPCR parallel to microscopy in the Amazon increases the observed prevalence from 38% to 64% (+26 (95%CrI 19 to 34) percentage points).

**Conclusion:**

The accuracy of either qPCR on DNA extracted from filter paper or microscopy for CL diagnosis as a stand-alone test seems to be unsatisfactory and region-dependent. We recommend further studies to confirm the clinically relevant increment found in the diagnostic yield due to the addition of qPCR.

## Author summary

Cutaneous leishmaniasis is caused by the parasite *Leishmania* and is treated when a microscopy test confirms the presence of the parasite in a sample of the lesion. This test, however, is known to miss patients with cutaneous leishmaniasis. DNA diagnostic tests (like PCR) that detect the parasite’s genetic material in the lesion, have been proposed to improve diagnosis. Filter paper preserves DNA at room temperature and allows samples to be transported from remote health centers to the PCR laboratory. The ability of microscopic and DNA testing to recognize leishmaniasis patients in real-life is complex to evaluate. We compared the performance of both tests using a statistical method that can evaluate both tests simultaneously without assuming that either test works perfectly. We found that PCR will be positive 68% of the time in a participant with leishmaniasis in the Amazon and 73% of the time in the Pacific region. In the Amazon, microscopy detects one out of every two cases, while it does in three out of every four cases in the Pacific. The addition of the PCR test can improve the number of participants with a diagnosis of leishmaniasis, mostly in the Amazon region.

## Introduction

### Background

Protozoan parasites of the genus *Leishmania* are the causative agent of cutaneous, mucosal, and visceral human leishmaniasis. The mainstay method for leishmaniasis confirmation is the combination of clinical characteristics and microscopy [1,2]. Worldwide, cutaneous leishmaniasis (CL) is the most common clinical manifestation of leishmaniasis affecting 600.000 to 1 million people annually [3]. CL manifests mainly as localized skin ulcers and nodules. In Ecuador it has an estimated prevalence of 3905–6415 cases or 30–49 per 100.000 inhabitants per year. It leads to an estimated health loss of 0.32 Disability Adjusted Live Years (DALY) per 100.000 people per year in the country and affects poor and indigenous populations disproportionally [4, 5].

According to the Ecuadorian Ministry of Health (MoH), direct microscopy observation of the parasite (smear or biopsy) is the “gold standard” to diagnose CL in Ecuador. Culture, serological and molecular tests are not available at public health centres as in most endemic regions [6]. The diagnostic accuracy of smear slide microscopy provided in Ecuador has however not been systematically evaluated. The existing small, limited number of peer-reviewed studies (of 14 to 90 participants) show low sensitivities ranging from 14% to 51% and specificities of near 100%, however based on a comparison to composite reference standards [7–11] which might give a biased result. Patients are nevertheless provided anti-leishmanial treatment for free by the MoH, conditional on having received a positive microscopy diagnosis [12]. Given the aforementioned estimated low sensitivity of the diagnostic test, this might leave several thousands of patients without treatment every year.

Molecular methods are promising for CL diagnosis because of their reported high diagnostic accuracy compared to microscopy [13]. Centralization of molecular tests would save costs, but transportation generally requires a cold chain that is not available. Hashiguchi *et al*. proposed filter paper imprints of CL lesions as a solution for the transport challenges [12]. Filter paper allows prolonged sample DNA conservation without the need for a cold chain [14]. An alternative approach to improve the diagnostic yield is the use of epidemiological, demographic, and clinical characteristics in the diagnostic process [6,15]. Weigle *et al*. reported high sensitivity of the use of a clinical diagnostic algorithm for Colombian CL, but the estimated specificity was low. A corresponding algorithm, however, has to be adapted and validated regionally before its implementation [16]. Of special clinical interest is the predictive value of the tests in use to evaluate the likelihood the participant has or does not have the disease after including the test result [17].

The lack of a reliable reference standard test or thus the absence of a true “gold standard” for CL diagnosis makes it challenging to assess the accuracy of any new diagnostic method and to investigate the true accuracy of the tests in use [18]. Two commonly proposed solutions to this problem are the use of 1) a composite reference standard, and 2) Bayesian Latent Class Analysis (LCA). Diagnostic test accuracy estimates based on a composite reference standard may be biased because combining multiple imperfect tests does not make one perfect reference standard [19], nor can the accuracy of the tests in the composite be evaluated. LCA, in comparison, applies a statistical model that simultaneously analyzes the results of the different tests observed while taking the imperfect nature of each test into account. Provided the assumptions of the model are correct, LCA allows unbiased estimation of the test accuracies, without depending on a perfect reference test [20,21].

In this prospective cross-sectional study, we used Bayesian LCA to estimate the diagnostic accuracy (sensitivity and specificity) of qPCR on DNA extracted from filter paper and microscopy for the diagnosis of CL in Ecuador. As a secondary objective, we assessed the predictive values of specific demographic and clinical criteria in our study population. These objectives are in accordance with the WHO Road Map for Neglected Tropical Diseases, 2021–2030 [22].

## Methods

### Ethics statement

This prospective study was approved by the ethical committee of the `Universidad Internacional del Ecuador’ (registration number: UIDE-FCM-EDM-COM-18-0069) and by the Ecuadorian MoH (registration number: MSPCURI000284-3). All participants signed a written consent and received free treatment for leishmaniasis according to the Ecuadorian MoH guidelines. The full study protocol can be accessed upon reasonable request to the corresponding author.

### Participants, data source, and data collection

This is a cross-sectional study of participants suspected of CL. Any case with a suspected cutaneous lesion for whom a physician practicing at a participating health center ordered CL testing was eligible for inclusion. Participants were excluded from the study if they did not provide written informed consent or had no cutaneous lesions. Participants were excluded from the diagnostic accuracy assessment if a qPCR and/or microscopy result was missing. Participants were included at three public primary health care centers in the Pacific subtropical region of the Pichincha province and from public and private primary health care centers and hospitals in the Napo, Pastaza, and Morona Santiago provinces in the Amazon (Total N = 16, See S1 Fig). Participants were identified and enrolled consecutively between the 1^st^ of January 2019 and the 30^th^ of June 2021 at the centers, and during community visits, by the physician, nurse, or laboratory technician, just before routine sampling for CL. The convenience sampling technique was used to obtain the study population sample size. A separate publication on this data provides additional details on the sample size calculation [23]. All centers had the laboratory capacity to perform smear slide microscopy and offered free treatment for CL-confirmed participants (intramuscular meglumine antimoniate for 20 consecutive days) or treatment for alternative diagnoses. The demographics of participants and clinical characteristics of cutaneous lesions were recorded before sampling. Age was recorded in years and ethnicity was based on participant self-reporting. The variable ethnicity was dichotomized for the analysis in: Amerindian (Amazon Kichwa, Shuar, Achuar, Shiwiar, Zapara, Andwa, or Waorani) and Mestizo (other than Amerindian). Gender was recorded as binary (male/female) and health-seeking delay was defined as the time since lesion onset as mentioned by the participant. The lesion type was classified by the professional into the categories i) ulcer, ii) nodule, and iii) other and presented in the manuscript as proportions of the total number of lesion types (total number of participants with known lesion type plus number of participants with mixed lesion types). The number of lesions was counted by the professional as the number of lesions separated by healthy skin. Body location was drawn on a person figure by the health professional. Body location of the lesion was divided into the categories ‘Head and neck’, ‘Trunk’, ‘Upper limbs’, and ‘Lower limbs’ for analysis. This variable was presented in the manuscript as proportion of the total number of body locations with lesions (total number of participants with known body location plus number of participants with lesions in multiple body locations). The geographical location of the participant was recorded to estimate the altitude of the place of infection in meters from the altitude of the airstrip of the nearest village (http://www.ais.aviacioncivil.gob.ec/) or with topographic-map.com (https://es-ec.topographic-map.com/maps/6ogw/Ecuador/) and was dichotomized as well in the Amazon and the Pacific region. Species determination was done by sequencing Cytochrome B and MPI as described elsewhere [23]. All the study data were collected on paper forms and entered into an electronic data capture system (https://www.castoredc.com/). Data entry was done in duplicate by two independent investigators and computer validated. We used the STARD-BLCM guidelines for the reporting of the study [24].

### Sample collection and diagnostic tests

The sample for microscopy was collected by scraping the inner border of the cutaneous lesion. If a participant had more than one lesion, the most recent one was sampled. If a participant presented mixed lesion types (i.e., ulcer and nodule) the ulcer was sampled. The resulting material was collocated on a glass slide and Giemsa stained following the Ecuadorian MoH guidelines [25]. Smear slide microscopy was performed by WHO-trained and experienced microscopists at the local center and sequentially at the central facility for all primary positive smears for diagnosis confirmation. The final results are reported as positive or negative (binary) because Ecuadorian laboratory technicians are not trained in grading parasite density [25]. A positive microscopy test meant it had been read positive on two occasions (on-site and at the central laboratory). Discordant results (positive on only one occasion) were regarded as negative. Technicians were aware of the clinical characteristics of the participants but unaware of the results of qPCR testing. Laboratory technicians were responsible for reporting adverse reactions during or after sampling to the principal investigator. To collect the sample for qPCR, the local laboratory technician pressed a filter paper (903 Protein Saver Card (Whatman, Newton Center, MA)) three times, for at least one second each, on the inner border of the suspected lesion, immediately after scraping for the smear slide. Filter papers were dried and sent under uncontrolled conditions to the research laboratory of the `Universidad de las Americas’ in Quito by canoe, plane, bus, private car, and/or postal service. They were stored at room temperature in the including centers, during transport, and in the research laboratory in Quito. DNA was extracted from the filter paper according to a Chelex and Proteinase K based protocol [26] that is non-time consuming and cheap and therefore preferable for resource-restricted settings [27,28]. A piece of 2*2mm with visible material was separated from the filter paper and placed in a sterile 1.5mL tube containing 200μL of 10% (wt/vol) Chelex 100 (Sigma-Aldrich, USA) and 10μL (≥4 units) of Proteinase K (Invitrogen, USA). Samples were vortexed for 5 min, incubated at 56°C for 60 min, and then at 96°C for 20 min. Finally, samples were centrifuged at 10,000 g (earth’s gravitational force) for 5 min, and the supernatant containing extracted DNA (approximately 150*μL)* was removed to another sterile tube. Extracted DNA was quantified with the NanoDrop 2000 Spectrophotometer (Thermo Scientific, USA). *Leishmania* DNA was detected by duplex real-time PCR (qPCR) of the *Leishmania* 18S ribosomal (rDNA) gene, which was validated for South American *Leishmania* species [29] and the human Tumor Necrosis Factor (hTNF) gene as internal control [30]. *Leishmania* rDNA and hTNF probes were labeled with FAM and HEX as reporters, respectively. The qPCR reaction was prepared with 2μL of extracted DNA and 13μL of master mix containing: 1X TaqMan Universal PCR Master Mix (Applied Biosystems, USA), 500nM of each primer, 250nM of each probe, and nuclease-free water to complete a final volume of 15μL. The reaction was run in a CFX96 Dx Thermal Cycler (Bio-Rad, USA) following this protocol: 95°C for 10 min; 45 cycles of 95°C for 15 sec and 58°C for 60 sec (detection). The last step was optimized with an alignment/extension temperature gradient maintaining the temperature with the highest fluorescence and lowest Ct values. Other conditions were according to the manufacturer’s recommendations. The detection limit of the qPCR reaction was determined directly on a 10-fold dilution series of a 441bp synthetic DNA fragment (IDT, USA) containing the *Leishmania* rDNA and hTNF target sequences and did not include DNA extraction. The DNA extraction and qPCR were repeated once when the hTNF Ct value was >32 or negative. Detection of amplified Leishmania rDNA resulted in defining the sample positive, except for Ct values ≥40, which were classified as negative. Results were considered invalid if *Leishmania* rDNA qPCR was negative and the hTNF probe did not amplify. The technicians that performed the qPCR in Quito were unaware of the clinical characteristics of participants and the microscopy results.

### Data analysis

Descriptive statistics (proportions, means, and medians, as appropriate) were used to describe the study population. We drew a directed acyclic graph (DAG) to illustrate the relation between the diagnostic tests that were used (i.e., smear slide microscopy and qPCR), the quantity of the pathogen itself, i.e., the amastigotes, or their genetic material (DNA) in the samples, and the target condition, being CL (S2 Fig). Based on this, we reasoned that the available data could be used to construct a two-class latent model with the two classes being “presence of CL” and “absence of CL”. We used Bayesian latent class models, adjusted for conditional dependence, to estimate the sensitivity and specificity of qPCR and microscopy for the diagnosis of CL. We also estimated the likelihood ratios, the prevalence of CL in the study population correcting for their diagnostic accuracy and comparing to the observed prevalence, and the predictive values (negative and positive) with their 95% credible intervals (95%CrI). We used an informative prior for the specificity of microscopy (Beta (99,1) distribution with median of 99% (95%CrI 96%, 100%)) based on the known high specificity of the microscopy results. We also used an informative prior for qPCR specificity (Beta (97,3) with median of 97% (95%CrI 93%, 99%)) based on available evidence [13]. We present the sensitivity and specificity for both tests separately by region, the Amazon, and the Pacific (Model 1), after noticing the significant difference in sensitivity values between the two regions, not reflected in the pooled estimates (S1 Table). Sequentially, and as part of our secondary aim, we assessed the impact on diagnostic accuracy of pre-defined covariates or thus within pre-specified subgroups, i.e., defined by the socio-demographic and clinical variables. More specifically we investigated if the covariate health-seeking delay (cut-off 4 weeks) was an effect measure modifier versus confounder for the accuracy between the regions. We, therefore, allowed the model to estimate the sensitivity by covariate level (Model 2), while allowing for conditional dependence among CL positive subjects, in the subpopulations and the pooled sample test specificities. We further assessed the impact on diagnostic performance within pre-specified subgroups, i.e., defined by age (cut-off 20 years of age), gender, altitude of infection (cut-off 500m), body location of lesion (head and neck versus other location), assessing the predictive values in the subgroups. It was hypothesized that sensitivity for a single test would be clinically insufficient (threshold set at 80%) and that the NPV, which is dependent on the prevalence, would differ by 20 percentage points between the two regions. Only participants with paired samples, i.e., both a valid result for qPCR and microscopy, were included in the diagnostic accuracy analysis. For all statistics, medians and 95% CrIs were reported. Statistical significance was determined by a CrI of differences not including 0. To conclude and as a comparison, the accuracy of qPCR (and its 95% confidence interval (CI)) was estimated using microscopy as the reference standard from a two-by-two contingency table by region. We used R version 4.0 (R Foundation for Statistical Computing, Vienna, Austria, 2020), more specifically for carrying out Bayesian inference on the latent class model we used the rjags package.

## Results

### Participants

**Fig 1:**
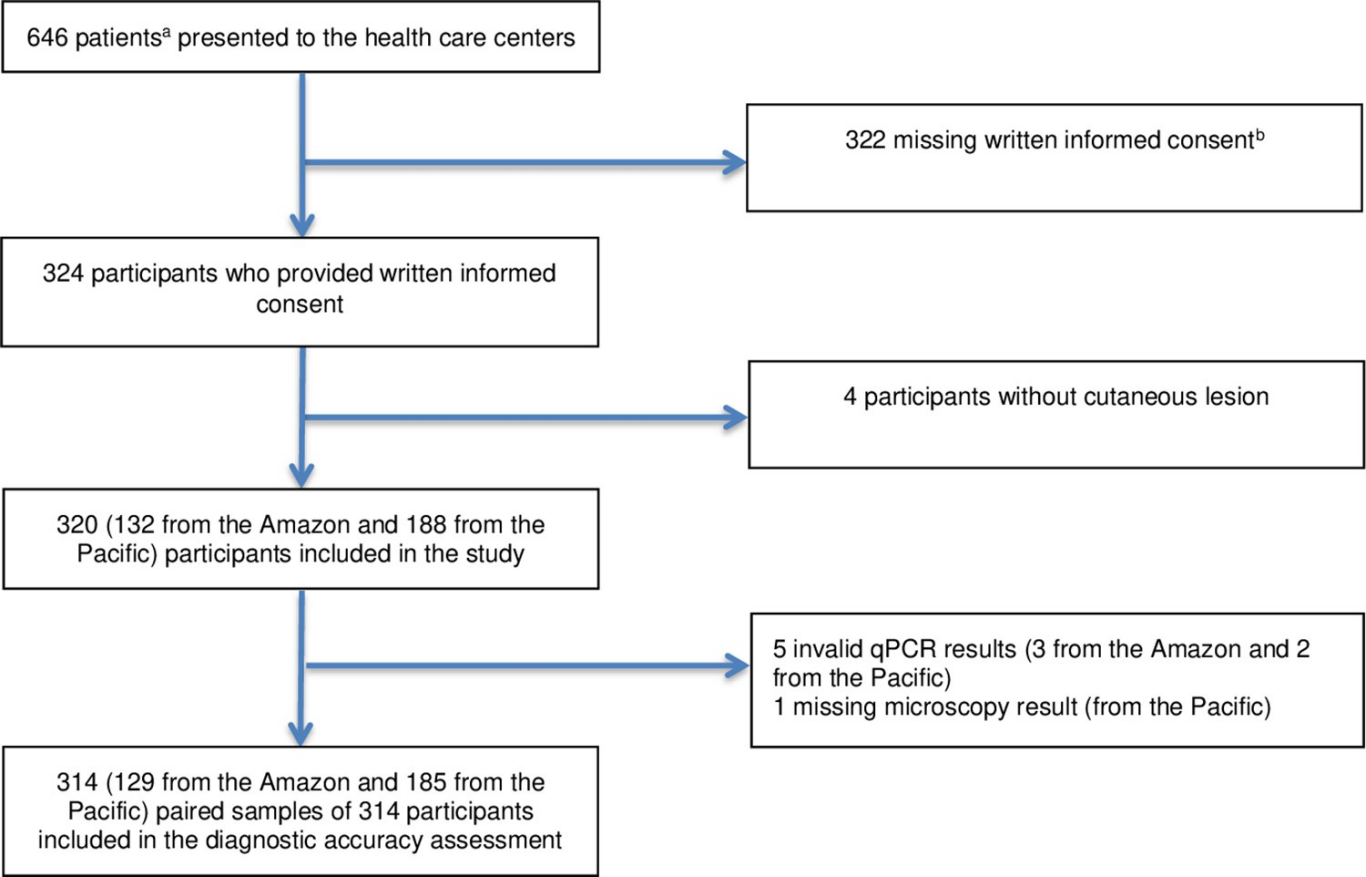
Flow chart of the study population. ^a^An eligible participant was defined as an individual presenting to one of the 16 participating healthcare posts with a cutaneous lesion instructed to sample for the diagnosis of cutaneous leishmaniasis. ^b^These patients decided not to participate in the study. Detailed and recorded reasons for this choice are not documented.

Participating centers enrolled 324 (50%) out of 646 eligible patients in the study (Fig 1). Four initially included participants were excluded because they had no cutaneous lesions. This resulted in a total of 320 included participants, a total of 188 (59%) participants from the Pacific and 132 (41%) participants from the Amazon region (Table 1). The mean age of the included participants was 26.8 years (range 0.1–88 years) and was higher in the Amazon (31.2 years) compared to the Pacific (23.7 years) region. A total of 100/188 (53%) participants included from the Pacific region were male compared to 85/132 (64%) from the Amazon. No Amerindian participants were included from the Pacific region compared to 87 (66%) from the Amazon. Median health-seeking delay in the Amazon was one month longer than in the Pacific (median 1 month). The lesion type (ulcer, nodule, or other) was known in 319/320 included participants who presented with a total of 322 lesion types. Three subjects presented with both ulcerated and nodular lesions and 16 with only nodular lesions. 290 participants presented with only ulcerated lesions and ten with only other lesion types. The median altitude of the presumed place of infection was 455m, which was similar in the Pacific and Amazon regions. CL suspected lesions were most frequently on the upper limbs (36%) and lower limbs (30%). Amazon participants had fewer lesions on the head and neck (13%) compared to Pacific participants (25%). None of the participants had initiated anti-leishmanial treatment prior to the sampling.

**Table 1:** Characteristics and clinical presentation of 320 suspected cutaneous leishmaniasis study participants from the Ecuadorian Pacific and Amazon regions by region and in total. Total number (%).

Participant characteristic (N missing for variables)	Pacific	Amazon	Total^c^
Number of Cases (%)	188 (59)	132 (41)	320 (100)
**General characteristics (1)**			
Mean age in years (SD)	23.7 (18.8)	31.2 (20.1)	26.8 (19.7)
Male (%)	100 (53)	85 (64)	185 (58)
Mestizo (%)	188 (100)	45 (34)	233 (73)
Amerindian (%)	0 (0)	87 (66)	87 (27)
**Clinical presentation (3)**			
Median health-seeking delay in months (IQR)	1.0 (0.5–2.0)	2.0 (0.9–4.0)	1.0 (0.5–2.0)
Total number of lesion types presented (%)^a^	189 (59)	133 (41)	322 (100)
Ulcer (%)	170 (90)	123 (92)	293 (91)
Nodule (%)	9 (5)	10 (8)	19 (6)
Other (%)	10 (5)	0 (0)	10 (3)
Median number of lesions (IQR)	1 (1–2)	1 (1–1)	1 (1–2)
Median altitude of suspected place of infection in meters above sea level (IQR)	532 (219–779)	396 (278–647)	455 (274–739)
**Body location of the lesion** ^**b**^			
Total number of body locations with lesions (%)	204 (59)	144 (41)	348 (100)
Head and neck (%)	51 (25)	18 (13)	69 (20)
Trunk (%)	22 (11)	25 (17)	47 (14)
Upper limbs (%)	77 (38)	49 (34)	126 (36)
Lower limbs (%)	54 (26)	52 (36)	106 (30)

N = Number, SD = Standard Deviation, IQR = Inter Quartile Range

^a^The denominator is lesion types. Lesion type was unknown in one participant. Participants with several lesion types were counted more than once.

^b^The denominator is body locations with lesions, participants with lesions on different body regions were counted more than once

^c^General description of 6 participants with missing paired samples is included in the table. 5 participants had an invalid PCR result, 1 had a lost microscopy sample. A total of 185 participants in the Amazon and 129 in the Pacific provided paired samples for the accuracy analysis.

### Laboratory results

All filter papers arrived at the research laboratory in Quito and qPCR was done for every participant. Samples of five participants had an invalid qPCR result. For one participant, microscopy slides got lost in the including laboratory and could not be recovered. The including centers reported no adverse events during or after the sampling of the specimens.

After exclusion of the above mentioned six participants with an invalid or missing qPCR or microscopy test result, 314 participants are included in the diagnostic accuracy analysis. A total of 186/314 (59%) qPCR samples tested positive (49% in the Amazon and 64% in the Pacific) compared to 176/314 (56%) microscopy positive samples. Discordant results, microscopy positive while qPCR negative, were present in 14% and 17% of the cases in the Amazon and the Pacific; microscopy negative while qPCR positive results were present in 26% and 15% in the same regions. Ct values by region and microscopy results are presented in S2 Table. The qPCR detected 1x10^-9^ ng/μL of synthetic DNA, which is equivalent to 4 copies of DNA in each 15uL reaction (S1 File). In 135/186 (73%) of the qPCR positive samples (71% Amazon, 74% Pacific) the causative *Leishmania* species could be determined, or in 46/129 (36%) and 89/185 (48%) of the total number of paired samples investigated in the Amazon and the Pacific, respectively [23]. Table 2 provides information on the species distribution by region, the sample internal control Ct values, and 18SrDNA Ct values by species. Microscopy positivity varied by species, with 84% of *L*. *guyanensis* positive and only 42% of *L*. *braziliensis* positive.

**Table 2:** Proportion microscopy positives by region and cycle threshold values of internal controls and of Leishmania 18rDNA in the 135 qPCR positive cases with *Leishmania* species identification, by species and in all samples combined.

	*L*. *guyanensis* (N = 102)^a^	*L*. *braziliensis* (N = 26)^a^	*L*. *lainsoni* (N = 7)^a^	All samples (N = 135)
**Microscopy positive (%)**				
**Total**	86 (84)	11 (42)	6 (86)	103 (76)
**Amazon**	13 (68)	7 (33)	5 (83)	25 (54)
**Pacific**^**b**^	73 (88)	4 (80)	1 (100)	78 (88)
**Median hTNF Ct (IQR)** ^**c**^	29.4 (27.3–31.0)	27.1 (25.7–28.9)	30.6 (29.9)	29.0 (26.8–30.8)
**Median *Leishmania* 18rDNA Ct (IQR)** ^**c**^	29.7 (27.7–32.5)	31.7 (29.8–35.2)	34.4 (27.7–35.3)	30.3 (27.8–33.3)

hTNF: human Tumor Necrosis Factor, Ct: Cycle Threshold, IQR: Interquartile Range

^a^ L*eishmania* species was determined in *Leishmania* 18SrDNA positive samples by sequencing a gene fragment that codes for the *Leishmania* Cytochrome B enzyme.

^b^By comparison, 81% (N = 83), 19% (N = 5) and 14% (N = 1) of the as *L*. *guyanensis*, *L*. *braziliensis*, *L*. *lainsoni* identified species in the total of investigated samples are from the Pacific region. A total of 66% of the identified species is from the Pacific region, with the remaining being from the Amazon.

^c^ hTNF was applied as internal control for sample taking and DNA extraction in a duplex qPCR together with *Leishmania* rDNA. Ct values have a logarithmic relationship with DNA concentrations and lower Ct values indicate higher DNA copy numbers.

### Diagnostic accuracy estimates of qPCR on DNA extracted from filter paper and microscopy

Applying our model (Model 1) to the population of the Amazon region, we found a sensitivity of qPCR of 68% (95%CrI 49;82) with a specificity of 97% (95%CrI 93;99), and a sensitivity of 51% (95%CrI 36;66) with a specificity of 99% (95%CrI 96;100) for microscopy. In the Pacific region, qPCR sensitivity and specificity were 73% (95%CrI 63;83) and 97% (95%CrI 93;99), respectively. Microscopy sensitivity was 76% (95%CrI 65;86) with a specificity of 99% (95%CrI 96;100) (Table 3). Microscopy sensitivity was statistically significantly lower in the Amazon compared to the Pacific (-24.9 percentage points (95%CrI -43.5; -6.7). Differences between qPCR and microscopy sensitivity were also largest in the Amazon (-16 percentage points (95%CrI -31;-2) and statistically significant. Estimating the diagnostic accuracy of qPCR and microscopy by health-seeking delay (Model 2) separately for the two regions, there is no evidence of an effect in the Amazon on qPCR or microscopy and a non-statistically significant effect on the microscopy in the Pacific region, with 8.1 percentage points (95%Cr -26.9;10.2) lower sensitivity in cases presenting more than 4 weeks after reported lesion appearance (Table 4). Using microscopy as the perfect reference standard, we estimated a sensitivity of 48% (95% confidence Interval (CI) 35;61) and specificity of 72% (95% CI 59;82) for qPCR in the Amazon and a sensitivity of 78% (95%CI 69;85) and specificity of 52% (95% CI 39;64) in the Pacific (Table 3).

**Table 3:** Diagnostic accuracy estimates with their 95% credible interval for qPCR and microscopy using latent class analysis and two-by-two table calculation (95% confidence interval).

	Model 1^a^ in subpopulation of the Amazon	Model 1^a^ in subpopulation of the Pacific	two-by-two-table in subpopulation of the Amazon^b^	two-by-two-table in subpopulation of the Pacific^c^
**Sensitivity qPCR**	68.0% (49.1;82.4)	73.4% (62.7;82.7)	63.3% (48.3;76.6)	75.2% (66.7;82.5)
**Sensitivity Microscopy**	51.2% (35.9;65.5)	76.4% (65.0;85.6)	-	-
***Difference sensitivity***^***d***^	-16.0 (-31.4;-1.7)	2.9 (-6.5;12.2)	-	-
**Specificity qPCR**	97.2% (92.6;99.4)	97.2% (92.8;99.4)	57.5% (45.9;68.5)	55.0% (41.6;67.9)
**Specificity Microscopy**	99.3% (96.1;100)	99.3% (96.1;100)	-	-
***Difference specificity***^***d***^	1.8 (-1.7;6.6)	1.8 (-1.7;6.4)	-	-
**LR+ qPCR**	24 (9;107)	27 (10;113)	1.5 (1.1;2.2)	1.7 (1.2;2.3)
**LR+ Microscopy**	71 (13;1852)	105 (19;2400)	-	-
**LR- qPCR**	0.3 (0.2;0.5)	0.3 (0.2;0.4)	0.6 (0.4;1)	0.5 (0.3;0.7)
**LR- Microscopy**	0.5 (0.3;0.6)	0.2 (0.1;0.4)	-	-

LR+: Positive Likelihood Ratio: true-positive proportion/false-positive proportion; LR-: false-negative proportion/true-negative proportion. A test with a LR+ of >10 is considered useful to rule in a diagnosis when a test is positive, while a test with a LR- <0.1 is considered useful to exclude a diagnosis when a test is negative.

^a^Latent class analysis with two latent classes, using the data of one joint population and informative prior for specificity microscopy with a beta distribution (99,1) and informative prior for specificity qPCR with a beta distribution (97,3). The model allows for conditional dependency between the two tests’ sensitivities and specificities.

^b^Two-by-two contingency table of samples from the Amazon region, using microscopy as the reference standard: true positives N = 31; false positives N = 34, false negatives N = 18, and true negatives N = 46.

^c^Two-by-two contingency table of samples from the Pacific region, using microscopy as the reference standard: true positives N = 94; false positives N = 27, false negatives N = 31 and true negatives N = 33.

^d^Difference between the estimate for qPCR and microscopy in percentage points

**Table 4:** Diagnostic accuracy estimates by delay in presentation for qPCR and microscopy using latent class analysis Model 2.

	Model 1^a^ in subpopulation of the Amazon	Model 1^a^ in subpopulation of the Pacific	two-by-two-table in subpopulation of the Amazon^b^	two-by-two-table in subpopulation of the Pacific^c^
**Sensitivity qPCR**	68.0% (49.1;82.4)	73.4% (62.7;82.7)	63.3% (48.3;76.6)	75.2% (66.7;82.5)
**Sensitivity Microscopy**	51.2% (35.9;65.5)	76.4% (65.0;85.6)	-	-
***Difference sensitivity*^*d*^**	-16.0 (-31.4;-1.7)	2.9 (-6.5;12.2)	-	-
**Specificity qPCR**	97.2% (92.6;99.4)	97.2% (92.8;99.4)	57.5% (45.9;68.5)	55.0% (41.6;67.9)
**Specificity Microscopy**	99.3% (96.1;100)	99.3% (96.1;100)	-	-
***Difference specificity*^*d*^**	1.8 (-1.7;6.6)	1.8 (-1.7;6.4)	-	-
**LR+ qPCR**	24 (9;107)	27 (10;113)	1.5 (1.1;2.2)	1.7 (1.2;2.3)
**LR+ Microscopy**	71 (13;1852)	105 (19;2400)	-	-
**LR- qPCR**	0.3 (0.2;0.5)	0.3 (0.2;0.4)	0.6 (0.4;1)	0.5 (0.3;0.7)
**LR- Microscopy**	0.5 (0.3;0.6)	0.2 (0.1;0.4)	-	-
	Model 1^a^ in subpopulation of the Amazon	Model 1^a^ in subpopulation of the Pacific	two-by-two-table in subpopulation of the Amazon^b^	two-by-two-table in subpopulation of the Pacific^c^
**Sensitivity qPCR**	68.0% (49.1;82.4)	73.4% (62.7;82.7)	63.3% (48.3;76.6)	75.2% (66.7;82.5)
**Sensitivity Microscopy**	51.2% (35.9;65.5)	76.4% (65.0;85.6)	-	-
***Difference sensitivity*^*d*^**	-16.0 (-31.4;-1.7)	2.9 (-6.5;12.2)	-	-
**Specificity qPCR**	97.2% (92.6;99.4)	97.2% (92.8;99.4)	57.5% (45.9;68.5)	55.0% (41.6;67.9)
**Specificity Microscopy**	99.3% (96.1;100)	99.3% (96.1;100)	-	-
***Difference specificity*^*d*^**	1.8 (-1.7;6.6)	1.8 (-1.7;6.4)	-	-
**LR+ qPCR**	24 (9;107)	27 (10;113)	1.5 (1.1;2.2)	1.7 (1.2;2.3)
**LR+ Microscopy**	71 (13;1852)	105 (19;2400)	-	-
**LR- qPCR**	0.3 (0.2;0.5)	0.3 (0.2;0.4)	0.6 (0.4;1)	0.5 (0.3;0.7)
**LR- Microscopy**	0.5 (0.3;0.6)	0.2 (0.1;0.4)	-	-

LR+: Positive Likelihood Ratio: true-positive proportion/false-positive proportion; LR-: false-negative proportion/true-negative proportion. A test with a LR+ of >10 is considered useful to rule in a diagnosis when a test is positive, while a test with a LR- <0.1 is considered useful to exclude a diagnosis when a test is negative.

^a^Latent class analysis with two latent classes, using the data of two subpopulations, i.e., the binary variable delay in presentation, allowing for differing sensitivities by stratum, and informative prior for specificity microscopy with a beta distribution (99,1) and informative prior for specificity qPCR with a beta distribution (97,3). The model allows for conditional dependency between the two tests sensitivities and between the subpopulation sensitivities, and two tests specificities.

^b^Difference between the estimate for qPCR and microscopy in percentage points

### Prevalence estimation, PPV, NPV, and effect of demographic and clinical characteristics on diagnostic performance

The prevalence of CL in the sampled cases was 73% (95%CrI 58;97) in the Amazon region and 88% (95%CrI 78;99) in the Pacific region using Model 1, correcting for test accuracy. In the Amazon, the addition of qPCR test positive cases to those identified by microscopy (observed prevalence microscopy positives: 38.0%) can increase the observed prevalence with 26.4 (95%CrI 19.3;34.4) percentage points. In the Pacific, the prevalence increases 14.6 (95%CrI 10.1;20.2) percentage points, also improving the diagnostic yield (Table 5). PPV and NPV are presented by region and by the different covariates age, altitude of infection, and lesion body location in with their estimates in the S3 Table, together with the estimates of covariate stratified sensitivities and specificities by region. NPV in the Amazon region was overall 54% (95%CrI 5;77) and 44% (95%CrI 4;65) for qPCR and microscopy respectively while being 34% (95%CrI 3;58) and 37 (95%CrI 3;63) in the Pacific region.

**Table 5:** Disease prevalence estimates in the sampled population correcting for the diagnostic accuracy of qPCR and microscopy (true prevalence); prevalence observed using qPCR and microscopy separately and the potential additional prevalence detected by adding qPCR positive cases to microscopy negative cases, by region.

	Amazon	Pacific
**Estimated true prevalence (Model 1)** ^**a**^	72.6% (57.9; 97.3)	87.7% (78.1;99.1)
**Observed prevalence of microscopy positive**	38.0% (30.0;46.6)	67.6% (60.6;74.0)
**Observed prevalence of qPCR positive**	50.4% (41.8;58.9)	65.4% (58.4;72.0)
**Observed prevalence of either microscopy or qPCR positive** ^**b**^	**64.3% (55.8;72.2)**	**82.3% (76.2;87.2)**
**Additional prevalence (in percentage points) diagnosed by adding qPCR** **to microscopy prevalence (microscopy negatives, qPCR positives)**	26.4 (19.3;34.4)	14.6 (10.1;20.2)

^a^The prevalence is estimated taking both test results and their imperfect accuracy into account and provides the estimated prevalence of CL disease, the target condition under investigation in the study sample.

^b^The sum is lower than the true prevalence estimates, given this is a prevalence based on observed positives and not corrected for imperfect accuracy. In this scenario, both false positive microscopy cases and false positive qPCR cases contribute to the prevalence and cases with positive qPCR and microscopy agreement are not double counted.

## Discussion

In this diagnostic accuracy study, using latent class analysis in participants presenting with suspected CL diagnosis in two regions in Ecuador, we found that the sensitivity of qPCR on DNA extracted from filter paper and smear slide microscopy is considerably lower in the Amazon region than in the Pacific region. This difference could not be explained by differences in health-seeking delay. In addition, different participants were diagnosed with either test. The specificity point estimates were 97% for qPCR and 99% for microscopy, using informative priors. The NPV reached its highest value in the lower prevalence region of the Amazon (prevalence 73%) both for qPCR and microscopy with a point estimate of 54% and 44% respectively, still lower than needed to confidentially consider the negative test as the proof of absence of CL in the participant. Adding the qPCR test and including qPCR positive cases as confirmed cases of CL would however increase the detection of cases with 15 to 26 percentage points, depending on the region. Other socio-demographic or clinical characteristics did not provide evidence to change the posterior probability of disease, more particularly, disease absence.

This study has several limitations. First, recruitment and thus the sample size suffered from the study being performed during the COVID-19 pandemic. During the COVID-19 pandemic, treatment supply at national level was interrupted temporarily and therefore the care for suspected CL patients was postponed in an unknown percentage. Additionally, individuals might have been afraid or had competing interests leading to lower participation rates than anticipated. We do not assume that participation was differential to qPCR or microscopy results, however. Because of the heterogeneous effect by region, the stratified sample sizes are relatively small, resulting in wider CrI. Secondly, with only two tests performed, we cannot further refine our model or assess the underlying mechanisms of the disagreement between the microscopy tests and qPCR. Third, the study population assessed might not be transportable to other environments, most specifically concerning their distribution of covariates. Additionally, the distribution of the covariates in the study population is conditional on the testing strategies that are in use in the different zones in Ecuador, which can lead to selection bias. Prevalence distributions can shift when the pre-test probabilities change over time or by center, alike the probability that an individual presenting with a lesion has an alternative diagnosis compared to CL. Fourth, the microscopic observations were made by different lab technicians leading possibly to a bias in the results, with most likely introduction of more false negatives, thereby decreasing the sensitivity of microscopy.

In this study, we aimed to answer a diagnostic question relevant to the correct diagnosis of CL when no good reference standard is available and where there is a lack of functional diagnostic tools for its diagnosis. The use of the LCA allowed the estimation of the sensitivity and specificity for qPCR and microscopy in the same data set, therefore estimating the accuracy of the test in use and the potential alternative or add-on test. By using an LCA we did not assume that microscopy has perfect sensitivity or specificity. We used priors for the specificity of both tests. While single microscopy tests can suffer from artifacts being recognized as amastigotes, the results, as in the current practice, are only positive when the presence of amastigotes is confirmed by the central laboratory, through second reading.

When compared to several other studies assessing the accuracy of PCR on DNA extracted from filter paper, our study found a lower sensitivity and lower positive agreement with microscopy [28,31]. On the other hand, our findings are consistent with a study of >700 Palestinian CL suspects that found a limited sensitivity of PCR on DNA extracted from filter paper and disagreement with smear slide microscopy [32]. Such a disagreement may be caused by a number of issues: First, because amastigotes are distributed unevenly across skin layers, the sample *Leishmania* DNA concentration may be affected by the sampling technique [33]. In our study, the sample for microscopy was obtained by scraping and the sample for qPCR by imprinting the lesion on filter paper. In other studies, lesion imprints on filter paper followed by PCR have shown better accuracy in comparison to scrapings followed by microscopy, however, also failed to detect *Leishmania* DNA in 8–17% of the proven microscopy positive samples [34–36]. A direct comparison of lesion imprints and scrapings on filter paper could reveal whether the sampling technique resulted in false negatives. Second, the sampling site may cause heterogeneity, but in this study, both the filter paper imprints and scrapings were taken from the lesion’s inner border [37]. Third, the handling and transport of the filter papers in remote tropical forest areas under uncontrolled conditions may still have affected the DNA quality. Fourth, when compared to other methods, the in our study used Chelex-based DNA extraction can result in more DNA extracted [38]. This may help detect *Leishmania* DNA, but abundant non-*Leishmania* DNA and contamination may also interfere with amplification [31,38]. Chelex has been applied in a limited number of studies to extract DNA from filter paper for the detection of CL, so an optimization study is recommended. Fifth, the *Leishmania* 18S qPCR used in this study has been validated in South American CL samples and is expected to detect the Ecuadorian endemic species [39]. Nonetheless, because the qPCR lacks a reverse transcriptase step, it does not amplify RNA, which may be more abundant in the samples than DNA. We recommend that in the future, the qPCR includes a reverse transcriptase step, as described by van der Meide *et al*. [29]. Finally, grading of the parasite density of *Leishmania* positive microscopy slides might have clarified an association between parasitemia and qPCR false negativity in our study and is recommended for future studies. We found heterogeneity in the sensitivity of microscopy by region in Ecuador. The country’s mainland is divided from north to south by the Andean highlands where CL is rare. CL clusters occur in the northwestern (Pacific) and the entire eastern (Amazon) region [4]. The probable reservoir, vector, and infecting *Leishmania* species differ by these regions. *L*. *guyanensis* is the prevalent species in participants from the Pacific region and a mix of *L*. *guyanensis*, *L*. *braziliensis*, *L*. *lainsoni*, and *L*. *naiffi* species is prevalent in the Amazon [4,12,40]. Detailed surveillance data by region are however not available. Additionally, participant presentation (age, health-seeking delay, and body location of lesions) and quality of life of CL-suspected patients are region dependent [5,12]. Participants from the Pacific region were included in the three cantons with Ecuador’s highest burden of CL (212–464 cases per 10.000 inhabitants). The Amazon participants were included from cantons with lower CL burden (17–212 cases per 10.000 inhabitants) [4]. The differences in participant presentation (lower age, shorter health-seeking delay, and more lesions on the head and neck in the Pacific region) and estimated CL prevalence as found in this study are in agreement with former publications [41,42]. However, this is the first publication to reflect on the diagnostic accuracy of both tests by region. The diagnostic program for leishmaniasis is Ecuador wide with uniform training procedures for microscopy technicians, as was the training of technicians involved in the filter paper sampling for this study [6]. Nevertheless, the lower prevalence of leishmaniasis in the Amazon may have resulted in fewer samples per technician, less experience, and, as a result, a lower sensitivity of smear slide microscopy [4]. Additional information about the lesions (diameter, wetness, infected or not) was not collected in this study, which could have helped to explain differences [43]. The causative *Leishmania* species, a prolonged period of health-seeking delay, and/or participant variables such as lesion location and age could all have influenced test performance [44]. This study’s strength is that it relied on existing diagnostic structures and thus reflects actual clinical practice. This has the limitation of not allowing us to draw conclusions about the determinants of test accuracies which should be addressed in future research. Because CL is more prevalent in the Pacific than in the Amazon, professionals in the Pacific (more experienced) may have sent fewer non-CL participants for *Leishmania* testing, resulting in a higher true prevalence. The differences in NPV depend on both test accuracy and estimated true prevalence and they remained below 80% in both the Amazon and Pacific, making it impossible to exclude CL after receiving a negative test from a suspected case.

The use of socio-demographic and clinical characteristics and their integration in the diagnostic pathway has been suggested, given this information is easily available also in very remote regions, is known on presentation, and does not require costly funding. Assessing the sensitivity and specificity of these characteristics, however, does not inform us how to interpret a positive or negative result of a qPCR or microscopy test and make a more informed treatment decision. Estimating the predictive values and likelihood ratios does allow their use in clinical decision-making. We showed that the sociodemographic and clinical characteristics as assessed do not improve the posterior probability to exclude the diagnosis of CL, i.e., the updated probability to not have the disease when the test is negative and this additional information is taken into account [45]. While, for example, exposure to the vector, i.e., in Ecuador the sandfly of the subfamily Phlebotominae genus *Lutzomyia*, has historically been defined by sex and/or gender, we did not see a difference in the prevalence of CL cases in males versus females who were tested for CL. As an additional remark, the covariates were dichotomized, which will result in information loss and might obscure real differences if we had chosen different cut-offs or had used the continuous variable.

Lessons learned from the results of this study for clinical practice and use of the diagnostic tests qPCR and microscopy are the following: First, a larger study is necessary to be able to decrease the uncertainty around the estimates. Second, data on the first microscopy and agreement with the confirmative reading will provide additional information. Our analysis can only be part of the general approaches to assess the true accuracy of tests used in the clinical diagnosis of CL. The addition and inclusion of direct PCR sampling and different sampling procedures can however optimize the specimen for PCR and its diagnostic yield, as is also the recommended diagnostic strategy as published in the IDSA guidelines [46]. Using microscopy solely for diagnosis maintains the status quo of under ascertainment and thus under treatment of participants with CL.

## Conclusions

The accuracy of the diagnostic tests evaluated for the diagnosis of CL seems to be unsatisfactory. Smear slide microscopy suffers from being insufficiently sensitive and being region dependent while having good specificity. None of the investigated tests, qPCR nor microscopy alone, has a sufficient performance to confidently rule out CL in the presenting participants. We recommend further studies on qPCR and microscopy accuracy to avoid increased morbidity and a sustained burden of DALY’s related to CL, due to participants who remained without a diagnosis and therefore untreated. An additional diagnostic test, either microscopy or qPCR, seems necessary to improve the overall sensitivity of the diagnostic strategy.

## Supporting information

S1 STARD-BLCM Checklist Completed STARD-BLCM Checklist. (DOCX)

S1 Fig Altitude map of Ecuador with geographic regions and participating health centers (N = 16). Black dots indicate major cities. Red dots indicate participating health center locations in the Pacific region: 1: Puerto Quito, 2: Pedro Vicente Maldonado, 3: San Miguel de Los Bancos, and in the Amazon region: 4: Tena Hospital, 5: Paushiyaku, 6: Satelital Tena, 7: Puerto Napo, 8: Misahualli, 9: Chontapunta, 10: Arosemena Tola, 11: Shell hospital, 12: Puyo hospital, 13: Tuutinentza, 14: Ipiak, 15: Wasakentsa, 16: Wachirpas. Copyright: The image is adapted from Wikipedia by the authors and is available under the Creative Commons CC0 1.0 Universal Public Domain Dedication [47].
(TIF)

S2 Fig Directed acyclic graph (DAG)—Graphical representation of the study question. The target condition under study is cutaneous leishmaniasis. Amastigotes or their genetic material (DNA) in the wound under suspicion are the measurands. The tests under evaluation are qPCR on DNA extracted from filter paper and direct microscopy after staining of a sample taken by wound scraping. The figure represents a DAG of the study question. We define two latent classes in this accuracy question, being (i) cutaneous leishmaniasis disease positive and (ii) disease status negative. Covariates potentially associated with a difference in prevalence of disease were assessed. Geographical region (Amazon versus Pacific region) was found an important factor. Other covariates were investigated separately by geographical region. Altitude of infection (500 m cut-off), body location of the lesion (head or neck versus elsewhere), health-seeking delay (cut-off 4 weeks) and age (younger or older than 20 years old) were only minimally different. Disease was equally distributed among both sexes.
(TIF)

S1 Table Diagnostic accuracy estimates for qPCR and microscopy with 95% credible Intervals using latent class analysis using region pooled data. LR: likelihood ratio; LR+: positive likelihood ratio; LR-: negative likelihood ratio. ^a^Latent class analysis with two latent classes, using the data of one joint population and informative prior for specificity microscopy with a beta distribution (99,1) and for specificity qPCR with a beta distribution (97,3). ^b^Difference between estimate for qPCR and for microscopy in percentage points
(DOCX)

S2 Table Characteristics of filter paper samples of 314 further analyzed cases. qPCR: quantitative Polymerase Chain Reaction, hTNF: human Tumor Necrosis Factor, Ct: Cycle Threshold, IQR: Interquartile Range. ^a^ hTNF was applied as internal control for sample taking and DNA extraction in a duplex qPCR together with *Leishmania* rDNA. Ct values have a logarithmic relationship with DNA concentrations and lower Cts indicate higher DNA copy numbers. ^b^ *Leishmania* species was determined in *Leishmania* 18SrDNA qPCR positive samples by sequencing a gen fragment that codes for the *Leishmania* Cytochrome B enzyme.
(DOCX)

S3 Table Prevalence, PPV and NPV with 95% credible Interval, by covariate using LCA^a^. ^a^Models use a beta distribution for the priors for sensitivity and specificity, including the informative prior for microscopy specificity of 99% and qPCR specificity of 97%.
(DOCX)

S1 File Limit of detection and standard curve of the qPCR reaction. (DOCX)

S2 File Study data of 320 participants. (XLSX)
