# Supplementary material for: Diagnostic accuracy of qPCR and microscopy for cutaneous leishmaniasis in rural Ecuador: A Bayesian latent class analysis
Source: PLoS Negl Trop Dis. 2023 Nov 29;17(11):e0011745. doi: 10.1371/journal.pntd.0011745 (PMC10686511; doi:10.1371/journal.pntd.0011745)
Supplement: S1 STARD-BLCM Checklist — (DOCX) [file pntd.0011745.s001.docx]

|  | **Section & Topic** | **No** | **Item** | **Reported on page #** |
| --- | --- | --- | --- | --- |
|  |  |  |  |  |
|  | **TITLE OR ABSTRACT** |  |  |  |
|  |  | **1** | Identification as a study of diagnostic accuracy, using at least one measure of accuracy (such as sensitivity, specificity, predictive values, or AUC) **and Bayesian latent class models**  “Diagnostic accuracy of qPCR and microscopy for cutaneous leishmaniasis in rural Ecuador: A Bayesian latent class analysis.” | 1 |
|  | **ABSTRACT** |  |  |  |
|  |  | **2** | Structured summary of study design, methods, results, and conclusions  (for specific guidance, see STARD for Abstracts)  The abstract is structured: **Background, Methods, Results, Conclusion.** More specifically the “**Methods:** This study compared the diagnostic accuracy of qPCR on DNA extracted from filter paper to the accuracy of direct smear slide microscopy in patients presenting with a cutaneous lesion suspected of leishmaniasis to 16 rural healthcare centers in the Ecuadorian Amazon and Pacific regions, from January 2019 to June 2021. We used Bayesian latent class analysis to estimate test sensitivity, specificity, likelihood ratios (LR), and predictive values (PV) with their 95% credible intervals (95%CrI) and assessed the diagnostic yield….” | 3 |
|  | **INTRODUCTION** |  |  |  |
|  |  | **3** | Scientific and clinical background, including the intended use and clinical role of the **tests under evaluation**  **Examples are: “**The mainstay method for leishmaniasis confirmation is the combination of clinical characteristics and microscopy [1, 2]….” And  “Patients are nevertheless provided anti-leishmanial treatment for free by the MoH, conditional on having received a positive microscopy diagnosis [12]. Given the aforementioned estimated low sensitivity of the diagnostic test, this might leave several thousands of patients without treatment every year.  Molecular methods are promising for CL diagnosis because of their reported high diagnostic accuracy compared to microscopy [13].” | 5,6 |
|  |  | **4** | Study objectives and hypotheses, **such as estimation of diagnostic accuracy of the tests for a defined purpose through BLCM**  “In this prospective cross-sectional study, we used Bayesian LCA to estimate the diagnostic accuracy (sensitivity and specificity) of qPCR on DNA extracted from filter paper and microscopy for the diagnosis of CL in Ecuador. As a secondary objective, we assessed the predictive values of specific demographic and clinical criteria in our population.” | 6,7 |
|  | **METHODS** |  |  |  |
|  | *Study design* | **5** | Whether data collection was planned before the **tests** were performed (prospective study) or after (retrospective study)  “In this prospective cross-sectional study” | 6 |
|  | *Participants* | **6** | Eligibility criteria **and description of the source population**  Specific paragraph: “**Participants, data source, and data collection”** and  “Any case with a suspected cutaneous lesion” | 7 |
|  |  | **7** | On what basis potentially eligible participants were identified  (such as symptoms, results from previous tests, inclusion in registry)  “for whom a physician practicing at a participating health center ordered CL testing” | 7 |
|  |  | **8** | Where and when potentially eligible participants were identified (setting, location and dates)  “Participants were included at three public primary health care centers in the Pacific subtropical region of the Pichincha province and from public and private primary health care centers” | 7 |
|  |  | **9** | Whether participants formed a consecutive, random or convenience series  “Participants were identified and enrolled consecutively” | 7 |
|  | *Test methods* | **10** | **Description of the tests under evaluation**, in sufficient detail to allow replication, **and/or cite references**  Complete section: **“Sample collection and diagnostic tests” –** including detailed description and references. | 8,9 |
|  |  | **11** | Rationale for choosing the **tests under evaluation in relation to their purpose**  “Given the aforementioned estimated low sensitivity of the diagnostic test, this might leave several thousands of patients without treatment every year.” And “Molecular methods are promising for CL diagnosis because of their reported high diagnostic accuracy compared to microscopy [13].” | 5,6 |
|  |  | **12** | Definition of and rationale for test positivity cut-offs or result categories of **the tests under evaluation**, distinguishing pre-specified from exploratory  “A positive microscopy test meant it had been read positive on two occasions (on-site and at the central laboratory).” And “Detection of amplified *Leishmania* rDNA resulted in defining the sample positive, except for Ct values ≥40, which were classified as negative.” | 8,9 |
|  |  | **13** | Whether clinical information was available to the performers or readers of **the tests under evaluation**  For microscopy: “Technicians were aware of the clinical characteristics of the patients but unaware of the results of qPCR testing.” And “The technicians that performed the qPCR in Quito were unaware of the clinical characteristics of patients and the microscopy results.” | 8,9 |
|  | *Analysis* | **14a** | **BLCM model** for estimating measures of diagnostic accuracy  “Based on this, we reasoned that the available data could be used to construct a two-class latent model with the two classes being “presence of CL” and “absence of CL”. We used Bayesian latent class models, adjusted for conditional dependence, to estimate the sensitivity and specificity of qPCR and microscopy for the diagnosis of CL.” And “ … We used an informative prior for the specificity of microscopy in (Beta (99,1) distribution with median of 99% (95%CrI 96%, 100%)) based on the known high specificity of the microscopy results. We also used an informative prior for qPCR specificity (Beta (97,3) with median of 97% (95%CrI 93%, 99%)) based on available evidence [13]. We present the sensitivity and specificity for both tests separately by region, the Amazon, and the Pacific (Model 1)..” | 10 |
|  |  | **14b** | **Definition and rationale of prior information and sensitivity analysis**  **Refer to 14a on prior information.**  **AND**  “We present the sensitivity and specificity for both tests separately by region, the Amazon, and the Pacific (Model 1), after noticing the significant difference in sensitivity values between the two regions, not reflected in the pooled estimates (S1 Table).”  And  “To conclude and as a comparison, the accuracy of qPCR (and its 95% confidence interval (CI)) was estimated using microscopy as the reference standard from a two-by-two contingency table by region.” | 11 |
|  |  | **15** | How indeterminate results **of the tests under evaluation** were handled  “Detection of amplified *Leishmania* rDNA resulted in defining the sample positive, except for Ct values >40, which were classified as negative.” | 10 |
|  |  | **16** | How missing data **of the tests under evaluation** were handled  “5 invalid qPCR results (3 from the Amazon and 2 from the Pacific)  1 missing microscopy result (from the Pacific)”-> (see flow diagram – missing data were not imputed but excluded from the analysis). | 12 |
|  |  | **17** | Any analyses of variability in diagnostic accuracy, distinguishing pre-specified from exploratory  “We further assessed the impact on diagnostic performance within pre-specified subgroups,…. ” | 10,11 |
|  |  | **18** | Intended sample size and how it was determined  “The convenience sampling technique was used to obtain the study population sample size was determined by convenience. ” | 7 |
|  | **RESULTS** |  |  |  |
|  | *Participants* | **19** | Flow of participants, using a diagram  “**Fig 1. Flow chart of the study population**” | 12 |
|  |  | **20** | Baseline demographic and clinical characteristics of participants  “**Table 1. Characteristics and clinical presentation of 320 suspected cutaneous leishmaniasis study participants from the Ecuadorian Pacific and Amazon regions**” | 12,13 |
|  |  | **21** | **Not applicable: the distribution of the targeted conditions is unknown, hence the use of BLCM** |  |
|  |  | **22** | Time interval and any clinical interventions between **the tests under evaluation**  “immediately after scraping for the smear slide.” | 9 |
|  | *Test results* | **23** | Cross tabulation of the **tests’ results (or for continuous tests results their distribution by infection stage)**  “two-by-two-table in subpopulation of the Amazon^b^” | 15 |
|  |  | **24** | Estimates of diagnostic accuracy **under alternative prior specification** and their precision (such as 95% **credible/probability intervals**)  “**Table 3: Diagnostic accuracy estimates with their 95% credible interval for qPCR and microscopy using latent class analysis and two-by-two table calculation (95% confidence interval)**” | 15,16 |
|  |  | **25** | Any adverse events from performing **the tests under evaluation**  “No adverse events took place during or after the sampling of the specimens.” | 13 |
|  | **DISCUSSION** |  |  |  |
|  |  | **26** | Study limitations, including sources of potential bias, statistical uncertainty, and generalisability  “This study has several limitations.” | 18-22 |
|  |  | **27** | Implications for practice, including the intended use and clinical role of **the tests under evaluation in relevant settings (clinical, research, surveillance etc.)**  **(related to if tests are NOT used):** “to avoid increased morbidity and a sustained burden of DALY’s related to CL, due to participants who remained without a diagnosis and therefore untreated” | 22 |
|  | **OTHER INFORMATION** |  |  |  |
|  |  | **28** | Registration number and name of registry  “(registration number: UIDE-FCM-EDM-COM-18-0069)” | 7 |
|  |  | **29** | Where the full study protocol can be accessed  “The protocol can be accessed upon reasonable request to the corresponding author.”  Note: As required by the Ecuadorian Ministry of Health, the protocol contains sensitive and private information on the investigators and participating institutions that cannot be made public. | 7 |
|  |  | **30** | Sources of funding and other support; role of funders  This is reported separately for PLOSNTD  Foundation Latin Link Nederland provided funding for the current study.  The funders had no role in study design, data collection and analysis, decision to publish, or preparation of the manuscript. |  |
|  |  |  |  |  |

STARD - BLCM

STARD-BLCM stands for “Standards for the Reporting of Diagnostic accuracy studies that use Bayesian Latent Class Models” and is a modification of the STARD statement (which was recently updated to STARD2015). STARD-BLCM aims to facilitate improved quality of reporting for diagnostic accuracy studies that use Bayesian latent class models in the absence of a reference standard. The proposed modifications are relevant to both Bayesian and frequentist estimation methods but the focus is on the former.

More information for STARD (STARD2015) can be found at: [http://www.equator-network.org/reporting-guidelines/stard](http://www.equator-network.org/reporting-guidelines/stard/)

More information for STARD-BLCM can be found at: [http://www.equator-network.org/reporting-guidelines/stard-blcm](http://www.equator-network.org/reporting-guidelines/stard-blcm/)
