# Supplementary figures and images for: Diagnostic accuracy of qPCR and microscopy for cutaneous leishmaniasis in rural Ecuador: A Bayesian latent class analysis
Source: PLoS Negl Trop Dis. 2023 Nov 29;17(11):e0011745. doi: 10.1371/journal.pntd.0011745 (PMC10686511; doi:10.1371/journal.pntd.0011745)

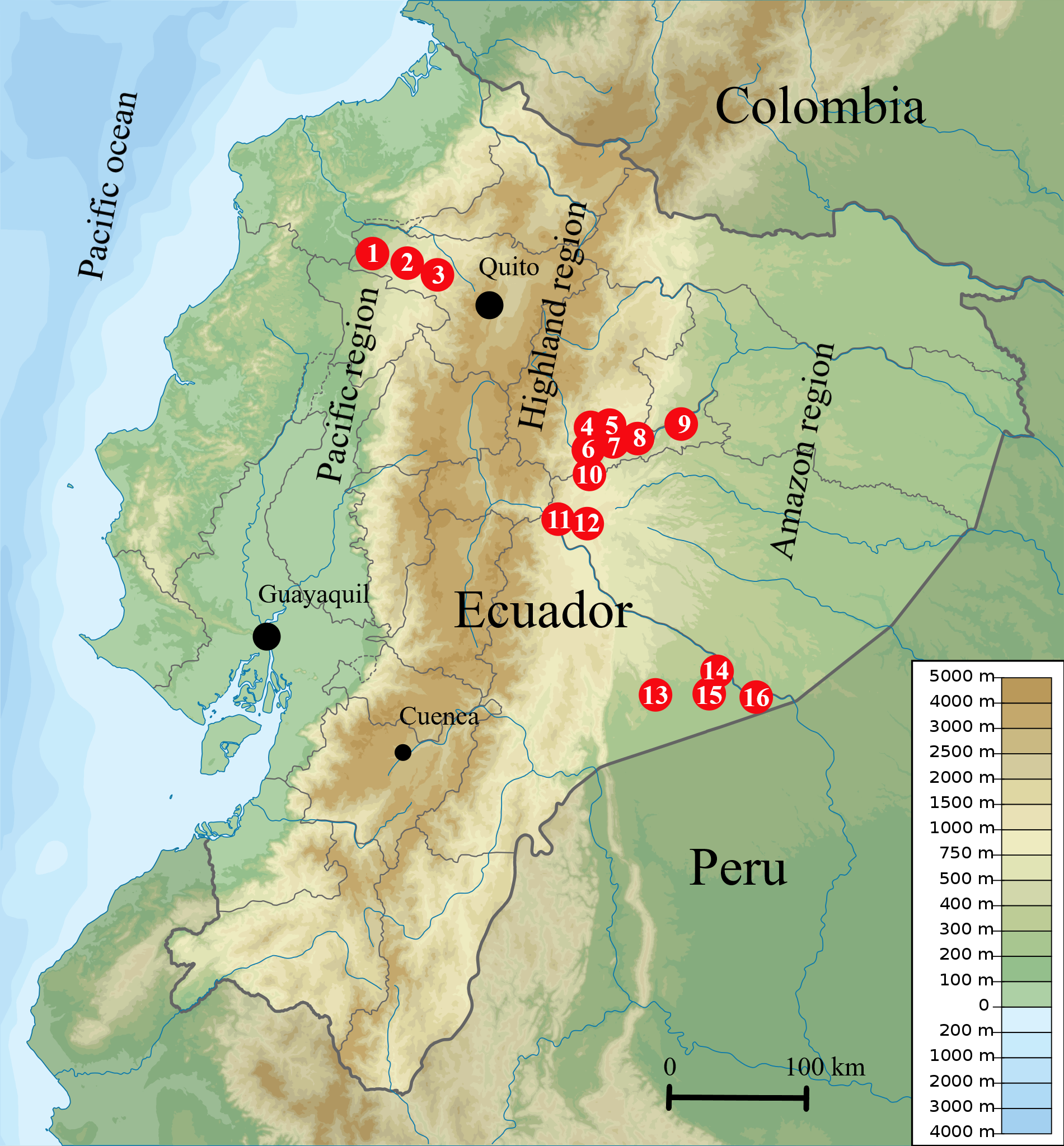

Supplement: S1 Fig — Black dots indicate major cities. Red dots indicate participating health center locations in the Pacific region: 1: Puerto Quito, 2: Pedro Vicente Maldonado, 3: San Miguel de Los Bancos, and in the Amazon region: 4: Tena Hospital, 5: Paushiyaku, 6: Satelital Tena, 7: Puerto Napo, 8: Misahualli, 9: Chontapunta, 10: Arosemena Tola, 11: Shell hospital, 12: Puyo hospital, 13: Tuutinentza, 14: Ipiak, 15: Wasakentsa, 16: Wachirpas. Copyright: The image is adapted from Wikipedia by the authors and is available under the Creative Commons CC0 1.0 Universal Public Domain Dedication [47]. (TIF) [file pntd.0011745.s002.tif]

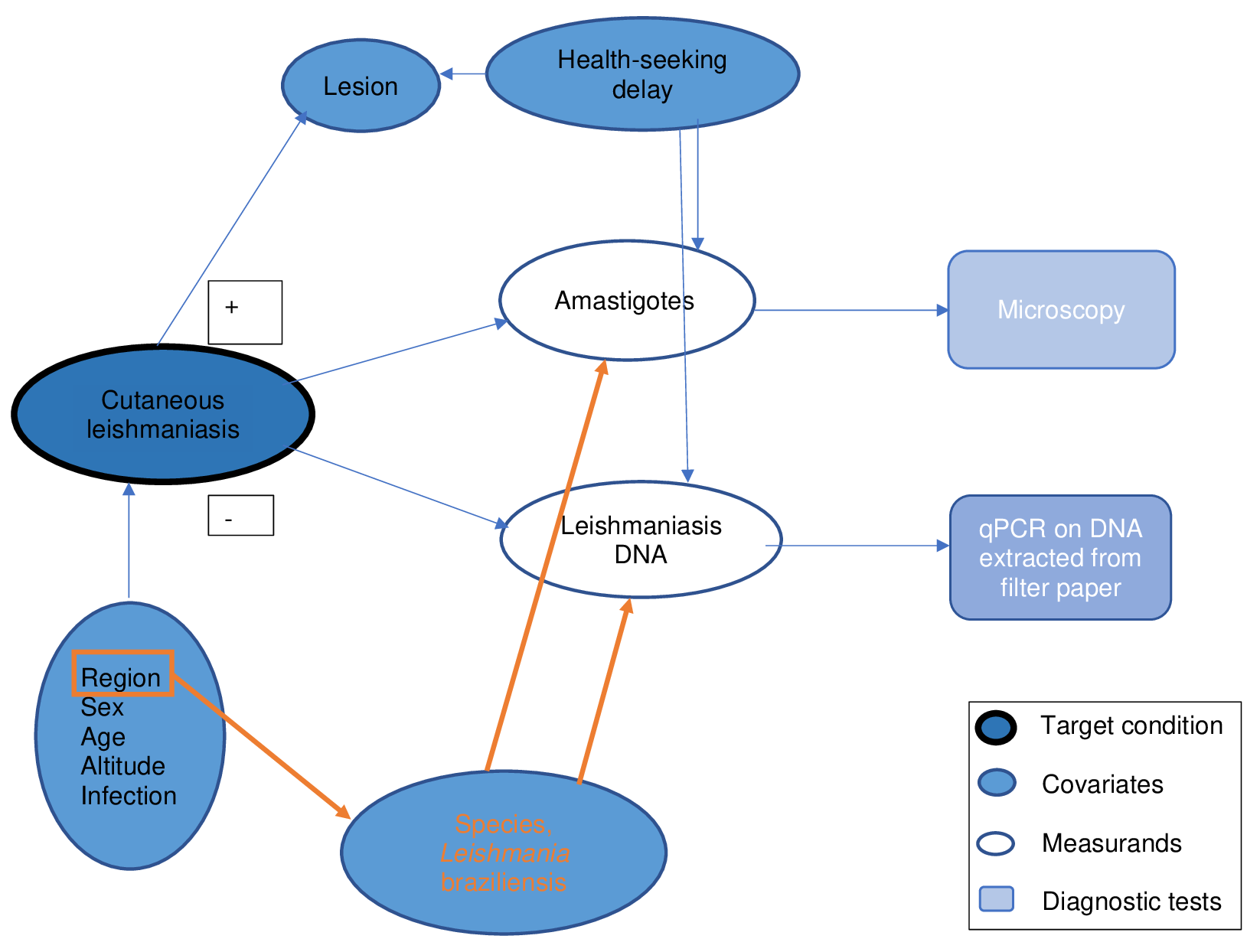

Supplement: S2 Fig — The target condition under study is cutaneous leishmaniasis. Amastigotes or their genetic material (DNA) in the wound under suspicion are the measurands. The tests under evaluation are qPCR on DNA extracted from filter paper and direct microscopy after staining of a sample taken by wound scraping. The figure represents a DAG of the study question. We define two latent classes in this accuracy question, being (i) cutaneous leishmaniasis disease positive and (ii) disease status negative. Covariates potentially associated with a difference in prevalence of disease were assessed. Geographical region (Amazon versus Pacific region) was found an important factor. Other covariates were investigated separately by geographical region. Altitude of infection (500 m cut-off), body location of the lesion (head or neck versus elsewhere), health-seeking delay (cut-off 4 weeks) and age (younger or older than 20 years old) were only minimally different. Disease was equally distributed among both sexes. (TIF) [file pntd.0011745.s003.tif]
