# Supplementary material for: Diagnostic accuracy of qPCR and microscopy for cutaneous leishmaniasis in rural Ecuador: A Bayesian latent class analysis
Source: PLoS Negl Trop Dis. 2023 Nov 29;17(11):e0011745. doi: 10.1371/journal.pntd.0011745 (PMC10686511; doi:10.1371/journal.pntd.0011745)
Supplement: S1 Table — LR: likelihood ratio; LR+: positive likelihood ratio; LR-: negative likelihood ratio. aLatent class analysis with two latent classes, using the data of one joint population and informative prior for specificity microscopy with a beta distribution (99,1) and for specificity qPCR with a beta distribution (97,3). bDifference between estimate for qPCR and for microscopy in percentage points (DOCX) [file pntd.0011745.s004.docx]

|  | Model 3^a^ |
| --- | --- |
| Sensitivity qPCR | 71.6% (58.5;81.3) |
| Sensitivity Microscopy | 67.2% (54.6;77.0) |
| *Difference sensitivity^b^* | -4.3 (-12.4;3.8) |
| Specificity qPCR | 97.3% (92.7;99.4) |
| Specificity Microscopy | 99.3 (96.4;100) |
| *Difference specificity^b^* | 1.9 (-1.4;6.5) |
| LR+ qPCR | 26 (9.5;112) |
| LR+ Microscopy | 98 (19;2624) |
| LR- qPCR | 0.3 (0.2;0.5) |
| LR- Microscopy | 0.3 (0.2;0.4) |
