# Supplementary material for: Diagnostic accuracy of qPCR and microscopy for cutaneous leishmaniasis in rural Ecuador: A Bayesian latent class analysis
Source: PLoS Negl Trop Dis. 2023 Nov 29;17(11):e0011745. doi: 10.1371/journal.pntd.0011745 (PMC10686511; doi:10.1371/journal.pntd.0011745)
Supplement: S2 Table — qPCR: quantitative Polymerase Chain Reaction, hTNF: human Tumor Necrosis Factor, Ct: Cycle Threshold, IQR: Interquartile Range. a hTNF was applied as internal control for sample taking and DNA extraction in a duplex qPCR together with Leishmania rDNA. Ct values have a logarithmic relationship with DNA concentrations and lower Cts indicate higher DNA copy numbers. b Leishmania species was determined in Leishmania 18SrDNA qPCR positive samples by sequencing a gen fragment that codes for the Leishmania Cytochrome B enzyme. (DOCX) [file pntd.0011745.s005.docx]

|  | **Pacific (N=185)** | **Amazon (N=129)** | **Microscopy positive AND *Leishmania* qPCR positive (N=125)** | **Microscopy negative AND *Leishmania* qPCR positive (N=61)** | **Microscopy positive AND *Leishmania* qPCR negative (N=49)** | **Time between sampling and DNA extraction >1 year (N=93)** | **All samples (N=314)** |
| --- | --- | --- | --- | --- | --- | --- | --- |
| **Median hTNF CT (IQR)^a^** | 30.0 (27.7-31.6) | 29.4 (28.0-31.6) | 29.5 (27.8-31.3) | 30.0 (28.0-32.2) | 30.7 (29.0-32.1) | 30.6 (28.7-32.4) | 29.8 (27.8-31.6) |
| **Median time in months between sampling and DNA extraction (IQR)** | 9.2 (7.7-11.8) | 6.2 (3.4-22.5) | 9.1 (5.0-11.8) | 8.7 (4.4-22.9) | 9.1 (7.7-19.7) | 23.3 (21.3-24.5) | 8.9 (4.7-20.5) |
| ***Total Leishmania* species determinations (%)^b^**  ***L. guyanensis* (%)**  ***L. braziliensis* (%)**  ***L. lainsoni* (%)** | 89 (48)  83 (93)  5 (6)  1 (1) | 46 (36)  19 (41)  21 (46)  6 (13) | 103 (82)  86 (83)  11 (11)  6 (6) | 32 (52)  16 (50)  15 (47)  1 (3) | 0 (0) | 29 (31) | 135 (43)  102 (76)  26 (19)  7 (5) |
| **Microscopy positive AND *Leishmania* qPCR negative (%)** | 31 (17) | 18 (14) | 0 (0) | 0 (0) | 49 (100) | 14 (15) | 49 (16) |
| **Microscopy negative AND *Leishmania* qPCR positive (%)** | 27 (15) | 34 (26) | 0 (0) | 61 (100) | 0(0) | 24 (26) | 61 (19) |
