# Supplementary material for: Diagnostic accuracy of qPCR and microscopy for cutaneous leishmaniasis in rural Ecuador: A Bayesian latent class analysis
Source: PLoS Negl Trop Dis. 2023 Nov 29;17(11):e0011745. doi: 10.1371/journal.pntd.0011745 (PMC10686511; doi:10.1371/journal.pntd.0011745)
Supplement: S3 Table — aModels use a beta distribution for the priors for sensitivity and specificity, including the informative prior for microscopy specificity of 99% and qPCR specificity of 97%. (DOCX) [file pntd.0011745.s006.docx]

|  | **Sensitivity qPCR/microscopy** | **Specificity qPCR/microscopy** | **Prevalence CL** | **PPV qPCR** | **PPV microscopy** | **NPV qPCR** | **NPV**  **microscopy** |
| --- | --- | --- | --- | --- | --- | --- | --- |
| **Amazon region** | 68.0 (49.1;82.4)/ 51.2 (35.9;65.5) | 97.2 (92.6;99.4) / 99.3 (96.1;100) | 72.6 (57.9; 97.3) | 98.7 (95.1; 99.9) | 99.6 (96.9; 100) | 54.0 (5.2; 77.4) | 43.9 (4.3; 64.5) |
| **Pacific region** | 73.4 (62.7;82.7) / 76.4 (65.0;85.6) | 97.2 (92.8;99.4) / 99.3 (96.1;100) | 87.7 (78.1; 99.1) | 99.6 (98.2; 100) | 99.9 (99.2; 100) | 34.1 (2.5; 57.5) | 37.3 (2.7; 62.6) |
| **Females**  **Amazon**  **Pacific** | 73.9 (51.9; 90.5) / 45.5 (28.7; 64.1)  68.7 (55.2; 80.9) / 73.4 (59.4; 85.4) | 97.2 (92.7; 99.3) / 99.3 (96.0; 100)  97.2 (92.6; 99.3) / 99.3 (96.2; 100) | 74.5 (55.1; 97.6)  87.0 (74.3; 99.1) | 98.9 (95.2; 99.9)  99.5 (97.7; 100) | 99.5 (96.3; 100)  99.9 (99.0; 100) | 57.1 (5.5; 85.7)  32.1 (2.3; 58.5) | 38.9 (3.8; 64.2)  36.2 (2.6; 65.3) |
| **Males**  **Amazon**  **Pacific** | 64.4 (44.6; 81.1) / 54.6 (36.9; 71.8)  76.7 (64.1; 87.1) / 78.3; 65.6; 88.7) | 97.2 (92.7; 99.3) / 99.3 (96.0; 100)  97.2 (92.6; 99.3) / 99.3 (96.2; 100) | 71.1 (54.3; 97.2)  88.1 (76.5; 99.1) | 98.5 (94.2; 99.9)  99.6 (98.2; 100) | 99.6 (96.6; 100)  99.9 (99.2; 100) | 53.4 (5.1; 77.7)  36.6 (2.8; 64.4) | 47.7 (4.6; 71.2)  38.7 (3.0; 68.3) |
| **<=20 years**  **Amazon**  **Pacific** | 75.4 (55.8; 90.2) / 44.9 (29.7; 61.4)  76.2 (63.6; 86.5) / 78.8 (66.1; 89.1) | 97.2 (92.7; 99.4) / 99.3 (96.1; 100)  97.2 (92.6; 99.4) / 99.3 (96.1; 100) | 79.8 (62.1; 98.3)  88.3 (77.1; 99.3) | 99.2 (96.5; 100)  99.6 (98.2; 100) | 99.7 (97.2; 100)  99.9 (99.2; 100) | 51.0 (4.3; 81.4)  35.6 (2.3; 62.9) | 31.7 (2.7; 55.8)  38.9 (2.5; 68.3) |
| **>20 years**  **Amazon**  **Pacific** | 62.2 (40.8; 80.0) / 57.1 (36.9; 75.6)  69.3 (55.6; 81.6) / 72.8 (58.5; 84.9) | 97.2 (92.7; 99.4) / 99.3 (96.1; 100)  97.2 (92.6; 99.4) / 99.3 (96.1; 100) | 66.8 (49.2; 96.3)  86.6 (73.2; 99.0) | 98.1 (92.6; 99.8)  99.5 (97.6; 100) | 99.5 (96.1; 100)  99.9 (98.9; 100) | 56.9 (6.5; 80.1)  35.6 (2.3; 62.9) | 54.3 (6.2; 77.6)  38.9 (2.5; 68.3) |
| **Infection altitude up to 500m**  **Amazon**  **Pacific** | 72.6 (54.5; 86.9) / 45.2 (31.3; 59.8)  72.7 (56.9; 85.7) / 68.9 (53.4; 82.4) | 97.1 (92.4; 99.3)/ 99.3 (96.3; 100)  97.2 (92.7; 99.3) / 99.3 (96.3; 100) | 77.9 (62.2; 98.0)  82.3 (68.5; 98.6) | 99.0 (96.1; 99.9)  99.3 (97.1; 100) | 99.6 (97.1; 100)  99.8 (98.6; 100) | 50.9 (47.6;78.1)  43.9 (3.5; 71.4) | 34.2 (3.2; 55.9)  41.2 (3.2; 68.2) |
| **Infection altitude >500m**  **Amazon**  **Pacific** | 59.2 (36.0; 80.2) / 64.1 (38.9; 86.8)  73.8 (63.0; 83.4) / 82.6 (71.5; 91.1) | 97.1 (92.4; 99.3)/ 99.3 (96.3; 100)  97.2 (92.7; 99.3) / 99.3 (96.3; 100) | 62.8 (42.7; 95.7)  92.1 (82.4; 99.5) | 97.6 (89.7; 99.8)  99.7 (98.7; 100) | 99.4 (95.9; 100)  99.9 (99.5; 100) | 59.5 (69.3;83.0)  24.3 (1.6; 49.3) | 63.1 (73.7; 88.5)  33.1 (2.1; 64.8) |
| **Head or neck lesion**  **Amazon**  **Pacific** | 42.4 (18.7; 71.2) / 70.6 (37.5; 95.5)  81.4 (67.3; 92.0) / 79.3 (65.1; 90.5) | 97.1 (92.4; 99.3) / 99.3 (96.2; 100)  97.2 (92.5; 99.3) / 99.3 (96.3; 100) | 61.7 (33.8; 95.3)  91.4 (78.7; 99.5) | 96.3 (79.5; 99.7)  99.7 (98.6; 100) | 99.4 (95.0; 100)  99.9 (99.4; 100) | 52.0 (67.6;80.7)  33.3 (2.2; 69.2) | 68.9 (8.9; 96.3)  31.5 (2.1; 66.3) |
| **Lesion other location than head or neck**  **Amazon**  **Pacific** | 72.3 (52.4; 86.5) / 49.2 (34.6; 63.5)  69.8 (57.6; 80.5) / 74.8 (61.9; 85.6) | 97.1 (92.4; 99.3) / 99.3 (96.2; 100)  97.2 (92.5; 99.3) / 99.3 (96.3; 100) | 73.3 (58.8; 97.4)  86.0 (74.6; 98.9) | 98.8 (95.4; 99.9)  99.4 (97.7; 100) | 99.6 (96.7; 100)  99.9 (99.0; 100) | 56.9 (55.6;80.6)  34.8 (2.9; 58.8) | 42.1 (41.4; 62.2)  39.6 (3.2; 66.4) |
| **Health-seeking delay**  **Amazon**  **Pacific** | 69.6 (45.6;87.8)/ 47.3 (29.1; 66.9)  72.7 (61.0;82.8) / 79.0 (66.8; 88.9) | 97.2 (92.7;99.4) / 99.3 (96.1;99.4)  97.2 (92.5;99.4) / 99.3 (96.4;100) | 68.5 (49.1;96.5)  88.4 (77.7; 99.2) | 98.4 (93.5; 99.9)  99.6 (98.1;100) | 99.4 (95.5; 100)  99.9 (99.2; 100) | 60.1 (6.7; 86.1)  32.7 (2.4; 57.5) | 46.9 (5.3;71.0)  39.5 (2.9; 68.0) |
| **No delay**  **Amazon**  **Pacific** | 66.7 (47.8;82.4)/ 54.7 (38.2;71.2)  74.2 (59;86.7) / 70.7 (55.5;84.2) | 97.2 (92.7;99.4) / 99.3 (96.1;99.4)  97.2 (92.5;99.4) / 99.3 (96.4;100) | 75.3 (58.5;97.7)  85.9 (71.8; 98.9) | 98.8 (95.2; 99.9)  99.5 (97.6; 100) | 99.6 (97.3; 100)  99.9 (98.9; 100) | 50.3 (5.0; 76.1)  38.5 (2.7; 68.8) | 42.8 (4.2; 66.9)  36.0 (2.6; 65.7) |
