# Supplementary material for: Diagnostic accuracy of qPCR and microscopy for cutaneous leishmaniasis in rural Ecuador: A Bayesian latent class analysis
Source: PLoS Negl Trop Dis. 2023 Nov 29;17(11):e0011745. doi: 10.1371/journal.pntd.0011745 (PMC10686511; doi:10.1371/journal.pntd.0011745)
Supplement: S1 File — (DOCX) [file pntd.0011745.s007.docx]

**Limit of Detection**

The limit of detection of the qPCR is 1x10^-9^ ng/µL of DNA after extraction, which is equivalent to 4.42 copies of DNA in each 15µL reaction.

| Starting Concentration of template [ng/µL] (copies/µL) | *Leishmania* Mean CT (SD) | Sensitivity of *Leishmania* DNA (detected/total) | hTNF Mean CT (SD) | Sensitivity of human DNA (detected/total) |
| --- | --- | --- | --- | --- |
| 1x10^-6^ (2210) | 27.4 (0.4) | 100% (3/3) | 29.7 (0.4) | 100% (3/3) |
| 1x10^-7^ (221.0) | 30.2 (0.3) | 100% (3/3) | 32.5 (0.2) | 100% (3/3) |
| 1x10^-8^ (22.10) | 33.6 (0.3) | 100% (3/3) | 35.9 (0.2) | 100% (3/3) |
| 1x10^-9^ (2.210) | 37.4 (0.2) | 100% (3/3) | 39.9 (0.4) | 100% (3/3) |

**Standard Curve**

The calibration curves of the two targets, *Leishmania* ribosomal DNA (LrDNA) and human tumor factor (hTNF), are shown in the graphic. On the y-axis, the CT value of the calibration curve points. On the x-axis, the logarithm of the initial DNA concentration according to the values of the standards.


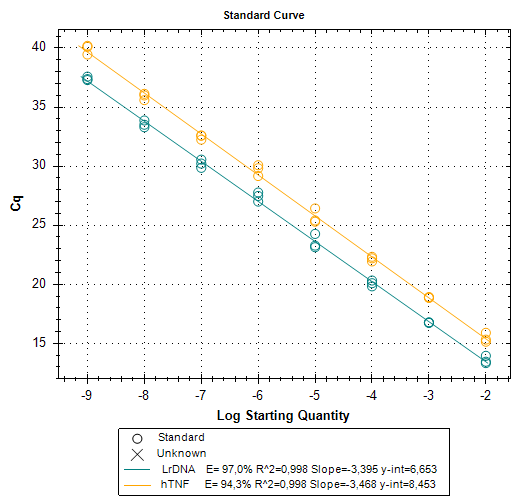


| Description | *Leishmania* DNA (LrDNA) | Human Tumor Necrosis Factor (hTNF) |
| --- | --- | --- |
| Slope (*m*) | -3.395 | -3.468 |
| y-int (*b*) | 6.653 | 8.453 |
| Efficiency (E) | 97% | 94.3% |
| r^2^ | 0.998 | 0.998 |
| Threshold Cycle (*CT*) | $CT=\left( -3.395 \right)*\log C+6.653$ | $CT=\left( -3.468 \right)*\log C+8.453$ |
| Quantification equation (*C*) | $C={10}^{\frac{CT-6.653}{-3.395}}$ | $C={10}^{\frac{CT-8.453}{-3.468}}$ |
